# Clinical evaluation of marketed and non-marketed orthodontic products: are researchers now ahead of the times? A meta-epidemiological study

**DOI:** 10.1186/s40510-023-00487-y

**Published:** 2023-10-23

**Authors:** Almaha Alhussain, Martyn T. Cobourne, Nikolaos Pandis, Jadbinder Seehra

**Affiliations:** 1 Department of Orthodontics, Faculty of Dentistry, Oral & Craniofacial Sciences, King’s College London, Floor 21, Guy’s Hospital, Guy’s and St Thomas NHS Foundation Trust, London, SE1 9RT UK; 2 Department of Orthodontics and Dentofacial Orthopedics, Dental School/Medical Faculty, University of Bern, Bern, Switzerland; 3 Centre for Craniofacial Development and Regeneration, Faculty of Dentistry, Oral & Craniofacial Sciences, King’s College London, Floor 27, Guy’s Hospital, Guy’s and St Thomas NHS Foundation Trust, London, SE1 9RT UK

**Keywords:** Marketed products, Orthodontics, Randomised clinical trials, Industry funding

## Abstract

**Background:**

The advertisement and adoption of untested orthodontic products is common. This study aimed to provide an update regarding the prevalence of clinical trials in orthodontics evaluating commercially marketed products. Associations between marketed/non-marketed products and study characteristics such as direction of effect, declaration of conflict of interest and industry sponsorship were evaluated. In addition, within the marketed products associations between direction of effect and study characteristics were explored.

**Material and methods:**

Electronic searching of a single database (Medline via PubMed) was undertaken to identify Randomized controlled trials (RCTs) published over a 5-year period (1st January 2017 to 31st December 2021). Descriptive statistics and associations between trial characteristics were explored.

**Results:**

196 RCTs were analysed. RCTs were frequently published in Angle Orthodontist (18.4%), American Journal of Orthodontics and Dentofacial Orthopedics (14.8%) and European Journal of Orthodontics (13.3%). 65.3% (128/196) of trials assessed marketed products after their introduction. The majority of trials assessed interventions to improve treatment efficiency (33.7%). Growth modification appliances were typically analysed in non-marketed compared to marketed products. An association between the type of product (marketed vs non-marketed) and both the declaration of conflict of interest and industry sponsorship was detected. For individual RCTs assessing marketed products either a positive effect (45.3%) or equivalence between interventions or between intervention and untreated control (47.7%) was evident. In 27% of these trials either no conflict of interest or industry funding was not clearly declared. Within the marketed products, no association between the direction of the effect and conflict of interest or funding was detected.

**Conclusions:**

The analysis of marketed orthodontic products after their introduction is still common practice. To reduce research waste, collaboration prior to the licensing and marketing of orthodontic products between researchers, industry and manufacturers is recommended.

## Introduction

For innovation to thrive in healthcare, clinicians should engage with industry and manufacturers to develop products which could benefit patients undergoing treatment. A successful example of this clinician-industry partnership is the creation of recombinant factor VIIa which was driven by the results from industry-led clinical trials [1]. Within the orthodontic speciality some companies do provide funding to support clinical research [2]. Nearly 38% orthodontic trials reported receiving funding or financial support, of which approx. 23% was from industry sources [3]. Despite, the obvious benefit to patient care, this engagement with industrial partners may be susceptible to both known and unknown bias which could compromise the validity of clinical trials [4]. This is highlighted by the results of a survey of scientists, in which 16% reported to have modified features of the study in response to pressure from funders [5]. Additionally, a clear association between industry funding and the publication of pro-industry results has been established [6, 7].

Within orthodontics, concerns regarding the advertisement and early adoption of relatively untested products have been raised [8]. In a large sample review of product advertisements published in orthodontic journals, 34.7% of these were supported by evidence. However, only 10.5% included accessible references [9]. More recently the claims made by marketed orthodontic products posted on social media have been reported in the main not to be supported by evidence and worryingly underpinned by false claims [10]. To circumvent this, an evidence-based approach considering both patient values and preferences to healthcare has been advocated. This should be underpinned by the results of high-quality studies such as randomized controlled trials (RCTs) which represent the gold standard for assessing both the effectiveness and safety of treatment interventions. However, RCTs can be costly and time-consuming. On this basis, to prevent research waste [11], the justification of an RCT should be supported by an appropriate systematic review of the current available literature [12].

A previous assessment of marketed orthodontic products reported that just under 50% of clinical trials published between 2012 and 2016 involved the analysis of these products after their introduction [13]. Therefore, this current study aimed to provide an update regarding the prevalence of clinical trials in orthodontics evaluating commercially marketed and non-marketed products. A secondary aim was to evaluate the presence of associations between the direction of the results of these trials (marketed vs non-marketed products) and both declaration of conflict of interest and industry sponsorship.

## Materials and methods

This study was reported in accordance with the guidelines for reporting meta-epidemiological methodology research [14]. The protocol for this study was not registered.

### Eligibility criteria

The methodology of this investigation is a replication of a previously published study [13]. English language randomized controlled trials (RCTs) published over a 5-year period (1st January 2017 to 31st December 2021) were considered for inclusion. Observational studies, editorials, letters, systematic reviews, commentaries, case reports, animal and laboratory studies were excluded. There was no restriction on regarding the type of product.

### Search for relevant articles

Electronic searching of a single database (Medline via PubMed) was undertaken on the following date: 7th January 2022. The term “orthodontics and Randomised Clinical Trials” was searched using the database filters. The term “Randomized Clinical Trials” was also searched with minimal difference in the number of articles identified. Based on the Cochrane criteria for the selection of RCTs, studies were screened for eligibility using the following criteria: human participants, interventions related to healthcare, experimental studies, presence of a control group and randomization of participants to control and treatment groups. Studies described in the title or abstract as “prospective”, “comparative”, or “efficacy” were further analysed to determine if randomization of participants was undertaken.

### Selection and data extraction

Two assessors (AA and JS) undertook independent screening of article titles, abstracts, and full texts. Any disagreements regarding the eligibility were discussed between both assessors and a third assessor (NP) until a consensus was reached. A standardized pre-piloted data extraction spreadsheet was used. Prior to data extraction, a pilot calibration between two assessors (AA and JS) was performed. 100% agreement was achieved. All data was then extracted independently by two reviewers (AA and JS). Any disagreements were discussed until a consensus and 100% agreement was achieved. If required, in the event of a disagreement a third assessor was consulted (NP).

At the study level the following characteristics were extracted: journal title, year of publication, number of authors (1–3, 4–6, > 7), continent of corresponding author (Europe, Americas and Asia and other), type of product (marketed or non-marketed), intervention type (orthodontic bracket, orthodontic archwire, removable appliance including sleep apnoea devices, non-surgical adjunctive, surgical adjunctive, oral health, orthodontic auxiliaries, materials, growth modification, medication (e.g. topical LA), retention, technology (e.g. mobile app, social media) and radiographs), justification of marketed intervention (accelerate treatment, aesthetics, reduce iatrogenic effects, retain tooth position, reduce pain, improve knowledge oral health, dental development, treatment efficiency, compliance and systemic effects), direction of intervention effect (positive effect compared to control, negative effect compared to control, no difference detected between interventions or between intervention and untreated control), declaration of conflict of interest (conflicts exist and declared, no conflicts to declare and not clearly declared) and declaration of industry funding (industry funded and declared, no industry sponsorship to declare, not clearly declared). Funding received from national societies, educational institutes or healthcare boards was not considered as industry funding. An assessment of the risk of bias of individual studies, summary measures, synthesis of results or additional analyses was not applicable to this study.

### Statistics

Descriptive statistics were calculated for each trial characteristic. Associations between individual RCTs and type of products (marketed and non-marketed), reported direction of intervention effect and declaration of conflict of interest and declaration of industry funding was undertaken using Fisher’s exact t test. Within the marketed products, association between direction of effect and study characteristics were explored using Fisher’s exact test. The level of statistical significance for all tests was pre-specified at 0.05. Statistical analyses were performed with STATA® version 17 software (Stata Corporation, College Station, TX, USA).

## Results

**Fig. 1:**
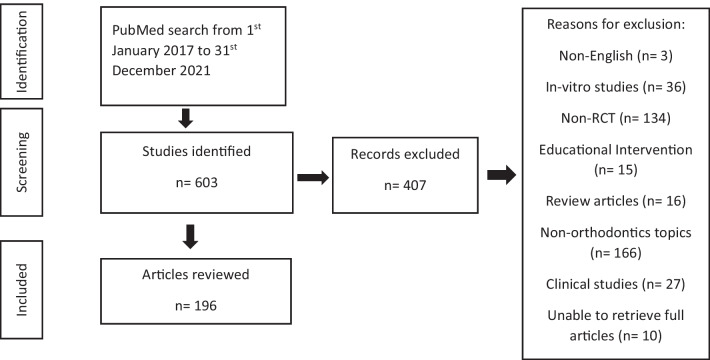
RCT identification flow diagram

Initial searching of the database yielded six hundred and three articles. Following application of the eligibility criteria, one hundred and ninety-six were included in the final analysis (Fig. 1). The number of RCTs published per year during the study timeframe (2017–2021) is shown in Table 1. In the final sample of one hundred and ninety-six RCTs, seven RCTs were published in 2022. However, these were identified in the initial search up until 31st Dec 2021 as early online publications and therefore were included in the analysis. The highest number of RCTs were published in Angle Orthodontist (18.4%), American Journal of Orthodontics and Dentofacial Orthopedics (14.8%) and European Journal of Orthodontics (13.3%) (Table 1).

**Table 1:** Journal titles included in analysis (N = 196)

Journal	N	Percent
Acta Odontologica Latinoamericana	1	0.51
Acta Odontologica Scandinavica	2	1.02
American Journal of Respiratory and Critical Care Medicine	1	0.51
American Journal of Orthodontics and Dentofacial Orthopedics	29	14.8
Angle Orthodontist	36	18.4
BMC Oral Health	4	2.04
BioMed Research International	1	0.51
Brazilian Dental Journal	1	0.51
Brazilian Oral Research	1	0.51
Caries Research	1	0.51
Clinical Oral Investigations	8	4.08
Clinical and Experimental Dental Research	1	0.51
Cranio	1	0.51
Dental Materials Journal	1	0.51
Dental Press Journal of Orthodontics	4	2.04
Dental and Medical Problems	2	1.02
European Journal of Paediatric Dentistry	1	0.51
European Journal of Orthodontics	26	13.3
International Journal of Dental Hygiene	1	0.51
International Journal of Environmental Research and Public Health	1	0.51
International Journal of Oral and Maxillofacial Surgery	1	0.51
International Journal of Paediatric Dentistry	2	1.02
International Orthodontics	7	3.57
Journal of Applied Physiology	1	0.51
Journal of Applied Oral Science	1	0.51
Journal of Clinical Sleep Medicine	5	2.55
Journal of Oral Science	1	0.51
Journal of Orofacial Orthopedics	8	4.08
Journal of Sleep Research	1	0.51
Journal of Investigative and Clinical Dentistry	1	0.51
Journal of the World Federation of Orthodontists	5	2.55
Laryngoscope	1	0.51
Lasers in Medical Science	4	2.04
Nigerian Journal of Clinical Practice	1	0.51
Photodiagnosis and Photodynamic Therapy	1	0.51
Photomedicine and Laser Surgery	1	0.51
The International Journal of Periodontics & Restorative Dentistry	1	0.51
International Journal of Dental Hygiene	1	0.51
Journal of Orthodontics	10	5.10
Journal of Clinical Sleep Medicine	1	0.51
Journal of Dentistry	1	0.51
Orthodontics & Craniofacial Research	7	3.57
Progress in Orthodontics	4	2.04
Scientific Reports	1	0.51
Sleep	1	0.51
Sleep and Breathing	3	1.53
Sleep Medicine	1	0.51
Thorax	1	0.51
Total	196	100.00

The majority of RCTs had 4–6 authors (53.6%) and had the corresponding author based in Europe (40.3%). 65.3% (128/196) of trials assessed marketed products after their introduction. Overall, commonly analysed interventions included growth modification (17.9%), materials (14.8%), removable appliances (14.3%) and non-surgical adjunctive (10.7%) (Table 2). Frequently, the justification of the intervention was to improve treatment efficiency (33.7%), reduce iatrogenic effects (24.5%), accelerate treatment (9.2%) and reduce pain (8.7%). In the overall sample of RCTs (n = 196), a positive effect compared to the control (44.9%) or no difference detected between interventions or between intervention and untreated control (47.4%) was reported. Although in the majority of RCTs (66.3%) no conflict of interest was declared, in nearly 30% it was not clearly declared (29.1%). A similar trend was also evident regarding the declaration of industry funding (30.1%) (Table 2).

**Table 2:** Trial characteristics (N = 196)

Trial characteristic	N (%)
*Year of publication*	
2017	37 (18.8)
2018	35 (17.9)
2019	36 (18.4)
2020	41 (20.9)
2021	40 (20.4)
2022	7 (3.6)
*Number of authors*	
1–3	43 (21.9)
4–6	105 (53.6)
> 7	48 (25.5)
*Continent of corresponding author*	
Europe	79 (40.3)
Americas	44 (22.5)
Asia and other	73 (37.2)
*Type of product*	
Marketed	128 (65.3)
Non-marketed	68 (34.7)
*Intervention type*	
Orthodontic bracket	13 (6.6)
Orthodontic archwire	9 (4.6)
Removable appliance	28 (14.3)
Non-surgical adjunctive	21 (10.7)
Surgical adjunctive	7 (3.6)
Oral health	11 (5.6)
Orthodontic auxiliaries	13 (6.6)
Materials	29 (14.8)
Growth modification	35 (17.9)
Medication (e.g. topical LA)	5 (2.5)
Retention	18 (9.2)
Technology (e.g. mobile app, social media)	6 (3.1)
Radiographs	1 (0.5)
*Justification of intervention*	
Accelerate treatment	18 (9.2)
Aesthetics	2 (1.0)
Reduce iatrogenic effects	48 (24.5)
Retain tooth position	18 (9.2)
Reduce pain	17 (8.7)
Improve knowledge Oral health	9 (4.6)
Dental development	7 (3.6)
Treatment efficiency	66 (33.7)
Compliance	9 (4.5)
Systemic effects	2 (1.0)
*Direction of intervention effect*	
Positive effect compared to control	88 (44.9)
Negative effect compared to control	15 (7.7)
No difference detected between interventions or between intervention and untreated control	93 (47.4)
*Declaration of conflict of interest*	
Conflicts exist and declared	9 (4.6)
No conflicts to declare	130 (66.3)
Not clearly declared	57 (29.1)
*Declaration of industry funding*	
Industry funded and declared	34 (17.4)
No industry sponsorship to declare	103 (52.5)
Not clearly declared	59 (30.1)
Total	196 (100.0)

Regarding interventions, orthodontic brackets (5.1%), removable appliances (9.7%), non-surgical adjunctive (8.2%) and materials (13.0%) were commonly assessed in marketed products compared to non-marketed products. In contrast, growth modification appliances (11.0%) were typically analysed in non-marketed compared to marketed products (Table 3). For individual RCTs assessing marketed products either a positive effect (45.3%) or equivalence between interventions or between intervention and untreated control (47.7%) was reported. Despite the majority of RCTs assessing marketed products declaring either no conflict of interest or industry funding, in equal numbers (26.6%) it was not clearly declared (Table 3). No significant associations between the direction of the intervention effect and type of product (marketed vs non-marketed) were evident (*p* = 0.92). Conversely, an association between the type of product (marketed vs non-marketed) and both declaration of conflict of interest (*p* = 0.05) and declaration of industry sponsorship (*p* < 0.001) was detected. Marketed products were more likely to declare a conflict of interest and industry sponsorship compared to non-marketed products (Table 4). For marketed products alone, no association was found between industry funding and direction of effect within the marketed products group (*p* = 0.32). Similarly, no association was found between conflict of interest and direction of effect (*p* = 0.92) (Table 5).

**Table 3:** Intervention types assessed for marketed and non-marketed products

	Product type		Total
	Marketed	Non-marketed	
Intervention type			
Orthodontic bracket	10 (5.1%)	3 (1.5%)	13 (6.6%)
Orthodontic archwire	6 (3.1%)	3 (1.5%)	9 (4.6%)
Removable appliance	19 (9.7%)	9 (4.6%)	28 (14.0%)
Non-surgical adjunctive	16 (8.2%)	5 (2.6%)	21 (11.0%)
Surgical adjunctive	4 (2.0%)	3 (1.5%)	7 (3.6%)
Oral health	9 (4.6%)	2 (1.0%)	11 (5.6%)
Orthodontic auxiliaries	6 (3.1%)	7 (3.6%)	13 (6.6%)
Materials	26 (13.0%)	3 (1.5%)	29 (15.0%)
Growth modification	14 (7.1%)	21 (11.0%)	35 (18%)
Medication	2 (1.0%)	3 (1.5%)	5 (2.6%)
Retention	12 (6.1%)	6 (3.1%)	18 (9.2%)
Technology	3 (1.5%)	3 (1.5%)	6 (3.1%)
Radiographs	1 (0.5%)	0 (0%)	1 (0.5%)
Total	128 (65.3%)	68 (34.7%)	196 (100%)

**Table 4:** Associations between marketed versus non-marketed products and direction of intervention effect, declaration of conflict of interest and declaration of industry funding

Characteristic	Marketed N (%)	Non-marketed N (%)	Fishers exact (*p* value)
*Direction of intervention effect*			
Positive effect compared to control	58 (45.3)	30 (44.1)	0.92
Negative effect compared to control	9 (7.0)	6 (8.8)	
No difference detected between interventions or between intervention and untreated control	61 (47.7)	32 (47.1)	
*Declaration of conflict of interest*			
Conflicts exist and declared	9 (7.0)	0 (0.0)	0.05
No conflicts to declare	85 (66.4)	45 (66.2)	
Not clearly declared	34 (26.6)	23 (33.8)	
*Declaration of industry funding*			
Industry funded and declared	33 (25.7)	1 (1.5)	< 0.001
No industry funding to declare	61 (47.7)	42 (61.8)	
Not clearly declared	34 (26.6)	25 (36.7)	
Total	128 (100.0)	68 (100.0)

**Table 5:** Within marketed products only, associations between direction of effect and study characteristics (Fisher’s exact test)

Variable	Negative versus control, N = 9^a^	No difference versus control/intervention, N = 61^a^	Positive versus control, N = 58^a^	p-value
Year				0.32
2017	3/9 (33%)	10/61 (16%)	7/58 (12%)	
2018	4/9 (44%)	11/61 (18%)	10/58 (17%)	
2019	0/9 (0%)	7/61 (11%)	16/58 (28%)	
2020	2/9 (22%)	14/61 (23%)	12/58 (21%)	
2021	0/9 (0%)	17/61 (28%)	11/58 (19%)	
2022	0/9 (0%)	2/61 (3.3%)	2/58 (3.4%)	
Author continent				0.24
Americas	1/9 (11%)	14/61 (23%)	12/58 (21%)	
Asia and other	5/9 (56%)	17/61 (28%)	22/58 (38%)	
Europe	3/9 (33%)	30/61 (49%)	24/58 (41%)	
COI				0.92
Conflict exists and declared	0/9 (0%)	5/61 (8.2%)	4/58 (6.9%)	
No conflicts to declare	7/9 (78%)	38/61 (62%)	40/58 (69%)	
Unclear	2/9 (22%)	18/61 (30%)	14/58 (24%)	
Funding				0.32
Industry funded and declared	2/9 (22%)	13/61 (21%)	18/58 (31%)	
Nothing to declare	5/9 (56%)	28/61 (46%)	28/58 (48%)	
Unclear	2/9 (22%)	20/61 (33%)	12/58 (21%)	

^a^n/N (%)

## Discussion

The conduct and reporting of clinical trials has been increasing within the orthodontic literature [15]. This is reflected by the fact that, compared to previous findings [13], a larger sample of RCTs was analysed in the current study. However, the majority of RCTs, 65.3% (n = 128) still assessed marketed products after their introduction. Furthermore, nearly 48% of trials reported no difference between interventions or between intervention and untreated control suggesting that a discord between marketing of products and assessing relevant clinical outcomes may exist. However, the lack of statistical difference between interventions does not always mean that the new product is worse than a gold standard intervention to which it is compared. In contrast, it may mean it is equally as effective as the comparison intervention, and this is clinically relevant. Conversely, approximately 45% of trials reported a positive effect compared to the control. The detection of initial significant differences for novel interventions is not uncommon in orthodontic trials and has been attributed to novelty bias [16]. Indeed, these initial exaggerated treatment effects are often subsequently not supported in the findings of future studies [16, 17]. This fact should be considered by clinicians when deciding when to introduce a new product into their clinical practice.

Consistent with previous findings [10, 13], trials tended to focus on interventions to improve treatment efficiency, reduce iatrogenic effects, accelerate treatment and reduce pain. It is reassuring that these outcomes appear to be patient centred [18]. Cumulative evidence from primary trials, suggests that surgical adjunctives may hasten treatment efficiency during particular stages of treatment [19, 20]. However, the methodological quality of these trials has been questioned and their effect on overall treatment duration is unclear. A key concern to patients during orthodontic treatment is potentially experiencing pain [21]. Recent systematic evidence supports that both pharmacological [22] and non-pharmacological [23] interventions may be beneficial in managing patients’ pain symptoms during orthodontic treatment. However, individually patients’ pain experience can be variable [24].

A conflict of interest (COI) has been defined as ‘a financial or intellectual relationship that may impact an individual’s ability to approach a scientific question with an open mind’ [25]. A potential COI could influence all facets of a study including the research question, study methodology, data analysis, selective reporting and interpretation of findings [26]. In this study, the majority of RCTs assessing marketed orthodontic products declared either no conflict of interest or industry funding. Interestingly, an association between the type of product (marketed vs non-marketed) and both a declaration of conflict of interest and declaration of industry sponsorship was evident. Within both medical and dental RCTs it is typical for any financial COI to be declared by the authors [27, 28]. However, in this sample in approximately 27% of RCTs the disclosure of conflict of interests or industry funding, was deemed to be not clear. This is consistent with previous dental literature, where in 32.5% of publications the presence of COI was unclear [28]. This lack of clarity regarding disclosure of COIs has been suggested to stem from a lack of awareness by researchers of the various forms of COI or the infrequencies of certain types of COI [27]. Generally, in the literature there is evidence of under-reporting of COIs by trial authors [29]. Importantly, a lack of clear reporting of COIs has been associated with trial misconduct [30].

It is disconcerting that marketed orthodontic products are advertised with limited supporting clinical evidence [9, 10]. As confirmed by previous studies [13] and the current investigation, the assessment of the clinical effectiveness of these products, tend to be the subject of clinical trials following their introduction which would appear to be a counter intuitive approach and not necessarily to the best interest of the patient. So, the question is which should come first “the chicken or the egg”. It is troubling that the industry can market and sell products for patients, often without the necessary evidence, and that researchers aiming to assess the effectiveness and safety of the product having to go through complicated and time-consuming processes just to get permission to test a product already sold and used [31]. As reported, 45% of trials reported equivalence in effectiveness between groups (another intervention or control). This may suggest in order to reduce research waste, and to ensure appropriate and relevant clinical outcomes are assessed, researchers should be involved earlier in the research and development process of orthodontic products [32]. However, the question remains if orthodontic industry and manufacturers have a similar desire to assess the effectiveness of their products prior to their introduction. Indeed, there may be some positive signs. Since 2010, Align Technology has been reported to provide a total of $2.7 million in funding to support research via their Research Award Programme [2]. However, this figure which translates to approximately $225,000 per year during this period, is dwarfed by the amount that this company spends annually on advisements and marketing for brand awareness [33]. To improve the reporting of COIs, trial authors should be given further guidance/explanation regarding the various types of COIs that exist with a clear distinction between financial, non-financial and sponsorship (non-profit and profit types) [27, 28]. This may allow journals to develop specific COIs forms which allow full disclosure by trial authors [28]. Trial authors could be also encouraged to register COI in registries which can be verified by the editorial teams of journals [27, 34]. Additionally, journal editorial teams can insist on complete reporting of trials in accordance with CONSORT checklist [27, 35]. However, within this checklist, item 25 pertains to the disclosure of financial COIs only. Hence in future updates of CONSORT the disclosure of non-financial COI should be included [28].

In this investigation, regardless of journal impact factor, orthodontic RCTs published in all journals were identified. We believe this approach allowed a better overall assessment of the publishing of trials assessing marketed products after their introduction. Non-English RCTs were excluded which may have resulted in potential selection bias. This source of bias may be further increased as only one database was searched. Furthermore, unlike previous investigations [27, 28], different types of COI were not explored in detail which may lead to under-estimation of the reported findings. To eliminate any other sources of bias, measures such as pre-piloting prior to data extraction and independent screening, selection, and data extraction by two reviewers was undertaken. Within the literature, it has been reported that the reproducibility of research study design is poor [36]. On this basis, we decided to adhere to the same methodology as previous investigations [13]. This facilitated comparison of the current results with those which have been previously published to allow us to determine the current prevalence of clinical trials in orthodontics evaluating commercially marketed products. It is acknowledged by the authors, that trials published in 2022–2023 were not included in this assessment. We feel this will not impact the reported results significantly as RCTs published within a 5-year timeframe (2017–2022) were included which allows assessment of current trends compared to the previously published data [13] which was based on RCTs published in 2012–2016.

## Conclusions

The analysis of marketed orthodontic products after their introduction is still common practice. These trials tend to focus on interventions to improve treatment efficiency, reduce iatrogenic effects, accelerate treatment and reduce pain. Growth modification appliances were typically analysed in non-marketed compared to marketed products. Marketed products were more likely to declare a conflict of interest and industry sponsorship compared to non-marketed products. Nearly 48% of trials reported no difference between interventions or between the intervention and untreated control. To reduce research waste, collaboration prior to the licensing and marketing of orthodontic products between researchers, industry and manufacturers is recommended.

## Data Availability

Not applicable.
